# Genome-Wide Genetic Diversity and Differentially Selected Regions among Suffolk, Rambouillet, Columbia, Polypay, and Targhee Sheep

**DOI:** 10.1371/journal.pone.0065942

**Published:** 2013-06-10

**Authors:** Lifan Zhang, Michelle R. Mousel, Xiaolin Wu, Jennifer J. Michal, Xiang Zhou, Bo Ding, Michael V. Dodson, Nermin K. El-Halawany, Gregory S. Lewis, Zhihua Jiang

**Affiliations:** 1 Department of Animal Sciences, Washington State University, Pullman, Washington, United States of America; 2 USDA/ARS US Sheep Experiment Station, Dubois, Idaho, United States of America; 3 Department of Dairy Science, University of Wisconsin-Madison, Madison, Wisconsin, United States of America; 4 Cell Biology Department, Division of Genetic Engineering and Biotechnology, National Research Center, Dokki, Gueza, Egypt; Auburn University, United States of America

## Abstract

Sheep are among the major economically important livestock species worldwide because the animals produce milk, wool, skin, and meat. In the present study, the Illumina OvineSNP50 BeadChip was used to investigate genetic diversity and genome selection among Suffolk, Rambouillet, Columbia, Polypay, and Targhee sheep breeds from the United States. After quality-control filtering of SNPs (single nucleotide polymorphisms), we used 48,026 SNPs, including 46,850 SNPs on autosomes that were in Hardy-Weinberg equilibrium and 1,176 SNPs on chromosome × for analysis. Phylogenetic analysis based on all 46,850 SNPs clearly separated Suffolk from Rambouillet, Columbia, Polypay, and Targhee, which was not surprising as Rambouillet contributed to the synthesis of the later three breeds. Based on pair-wise estimates of *F*_ST,_ significant genetic differentiation appeared between Suffolk and Rambouillet (*F*_ST_ = 0.1621), while Rambouillet and Targhee had the closest relationship (*F*_ST_ = 0.0681). A scan of the genome revealed 45 and 41 differentially selected regions (DSRs) between Suffolk and Rambouillet and among Rambouillet-related breed populations, respectively. Our data indicated that regions 13 and 24 between Suffolk and Rambouillet might be good candidates for evaluating breed differences. Furthermore, ovine genome v3.1 assembly was used as reference to link functionally known homologous genes to economically important traits covered by these differentially selected regions. In brief, our present study provides a comprehensive genome-wide view on within- and between-breed genetic differentiation, biodiversity, and evolution among Suffolk, Rambouillet, Columbia, Polypay, and Targhee sheep breeds. These results may provide new guidance for the synthesis of new breeds with different breeding objectives.

## Introduction

During the last five years, the animal genome community has made significant progress in mapping, sequencing, assembly, and annotation of the ovine genome. Based on BAC (bacterial artificial chromosome) end sequences, Dalrymple and colleagues [1] first reported a virtual sheep genome by painting a total of 84,624 sheep BACs (about 5.4-fold genome coverage) to orthologous regions in the human genome, which were assembled into 1,172 sheep BAC comparative genome contigs that covered 91.2% of the human genome. In 2009, Goldammer and coworkers [2] constructed a cytogenetic map of the sheep genome with 566 loci, which helped link and order genome regions, such as sequence contigs, genes, and polymorphic DNA markers to ovine chromosomes. Approximately two years ago, the International Sheep Genomics Consortium (ISGC) began assembly of a draft reference genome of sheep (*Ovis aries*) using both Sanger sequencing and the next-generation sequencing platforms [3]. This large scale sequencing of the ovine genome led to discovery of more than 2.8 million ovine single nucleotide polymorphisms (SNPs; http://www.ncbi.nlm.nih.gov/SNP/). In collaboration with the ISGC, Illumina developed the OvineSNP50 Genotyping BeadChip that contains a total of 54,241 SNPs with a marker placed approximately every 46 Kb along the sheep genome (www.illumina.com).

The Illumina OvineSNP50 Genotyping BeadChip has been successfully used in sheep and goat genome research. For example, BeadChip analysis revealed that the *PITX3* gene is responsible for microphthalmia [4]. A similar approach also helped identify the dentin matrix protein 1 gene (*DMP1*) as responsible for inherited rickets in Corriedale sheep [5] and the solute carrier family 13 (sodium/sulphate symporters), member 1 (*SLC13A1*) gene for chondrodysplasia in Texel sheep [6]. Both OvineSNP50 BeadChips and microsatellite markers were used to refine two quantitative trait loci (QTL) mapped on OAR5 and 13 for resistance to *Haemonchus contortus* in sheep [7]. Other applications of OvineSNP50 BeadChip include investigating gene drivers of pigmentation in Merino sheep [8], long range linkage disequilibrium analysis in wild sheep [9], inbreeding coefficient and pairwise relatedness in Finnsheep [10], and genomic selection in different sheep breeds from around the world by the ISGC [11].

It is well known that Suffolk and Rambouillet were developed in England and France, respectively, but the breeding history of American synthetic breeds may be unfamiliar to readers. In brief, Columbia was one of the first breeds of sheep developed in the United States. In 1912, rams of the long wool breeds were crossed with high quality Rambouillet ewes to produce large ewes yielding more pounds of wool and more pounds of lamb. The original cross was made at Laramie, Wyoming, and then moved to the Sheep Experiment Station, Dubois, Idaho, in 1918. Subsequently, Columbia sheep were released to the public [12]. Polypay sheep were developed at the U.S. Sheep Experiment Station starting in 1968. The objective was to develop a breed with a reproductive capacity markedly superior to that of domestic Western range breeds. The final composition of the Polypay is 1/4 Dorset × 1/4 Finnsheep × 1/4 Targhee × 1/4 Rambouillet. The first “Polypay” ewes and rams were sold 1975–1977 [13]. Targhee sheep were developed at the U.S. Sheep Experiment Station, Dubois, Idaho in 1926. A group of cross-bred ewes, consisting of Rambouillet, Lincoln, and Corriedale blood, was bred to USSES Rambouillet rams. After three years, first generation ewes were carefully selected and bred intensely. The U.S. Targhee Sheep Association was founded in 1951 (http://www.ustargheesheep.org/).

Generally speaking, sheep breeds can be classified into three groups: meat, wool, or dual-purpose breeds based on their breeding objectives. For example, Suffolk is a typical meat breed as the animals possess large body size, rapid growth rate, and high cutability carcasses (http://u-s-s-a.org/). On the other hand, Rambouillet sheep represent a fine wool breed with a well-developed flocking instinct, an extended breeding season, and high-quality fleece (http://www.countrylovin.com/ARSBA/facts.htm). Columbia, Targhee, and Polypay are, however, considered as dual-purpose breeds, because they are fast-growing, high-quality market lambs that also yield heavy, medium-wool fleeces with good staple length [12]–[13] (http://www.sheepusa.org/).

Previously, microsatellite markers were the main source of markers used to investigate genetic diversity of sheep breeds. For instance, Bayesian cluster analysis on microsatellite genotypes of 666 animals for 28 U.S. sheep breeds derived from 222 producers located in 38 states was able to distinguish meat vs. wool producers due to physiological differences rather than geographic origin [14]. In the present study, our goal was to test the power of the Illumina OvineSNP50 Genotyping BeadChip in evaluating genetic diversity, genome selection, and breed differentiation among Suffolk, Rambouillet, Columbia, Polypay, and Targhee sheep breeds. These results may provide new guidance for the synthesis of new breeds with different breeding objectives.

## Results

### Illumina OvineSNP50 BeadChip Genotyping Basics

Among the 54,241 SNPs on the Illumina OvineSNP50 BeadChip that were genotyped on the 94 sheep DNA samples, we observed that 695 SNPs had no calls, 1,019 SNPs were not genotyped for at least 95% of all the individuals, 1,235 SNPs were monomorphic in all breeds, 350 SNPs could not be assigned to chromosome locations and 2,057 SNPs had MAF ≤0.05 for the whole dataset. By excluding the SNPs described above, the remaining 48,885 SNPs, including 47,597 autosomal SNPs and 1,288 SNPs on chromosome X, were used for further analysis. Of the 1,288 SNPs on chromosome X, 1,176 SNPs identified non-heterozygous males, while 112 SNPs were heterozygous in some rams. This might be related to a homologous region between chromosomes X and Y because our data do not show that the heterozygous regions are random. As suggested by Gautier et al [15], we further excluded a total of 747 autosomal SNPs, which showed significant (*P*<0.01) deviations from the Hardy-Weinberg equilibrium (HWE) test due to the small number of samples. We did not test chromosome X because the SNPs on chromosome X in males carry only one copy. As a consequence, 46,850 autosomal SNPs were included in linkage disequilibrium, genetic diversity and DSR analyses, while the 1,176 SNPs with non-heterozygous males on chromosome X were used for DSRs analysis only. As shown in Figure S1, the final 46,850 and 1,176 SNPs were uniformly distributed on different autosomes (1–26) and the X chromosome, and were comparable to the initial distribution of the 54,241 SNPs on these chromosomes, although the numbers of SNPs in each chromosome were different between them.

### r^2^ Measurements by Chromosomes

The r^2^ values for pairs of loci were measured along with the physical distance separating the loci and averaged within each breed. As the sheep genome is currently estimated to be 2.86 Gb in size (http://genome.ucsc.edu/), the 46,850 SNPs used in linkage disequilibrium (LD) analysis would have an average inter-marker distance of approximately 60 Kb. As shown in Figure S2, the average within-population pairwise r^2^ dropped quickly toward its asymptotic value when physical distances reached 200 Kb. More interestingly, the decreasing trends of the average r^2^ values remained similar among these five breeds, but the Suffolk breed had the highest r^2^, followed by Columbia, Rambouillet, Targhee, and Polypay, respectively.

### The Genetic Structure at the Individual Level

**Figure 1:**
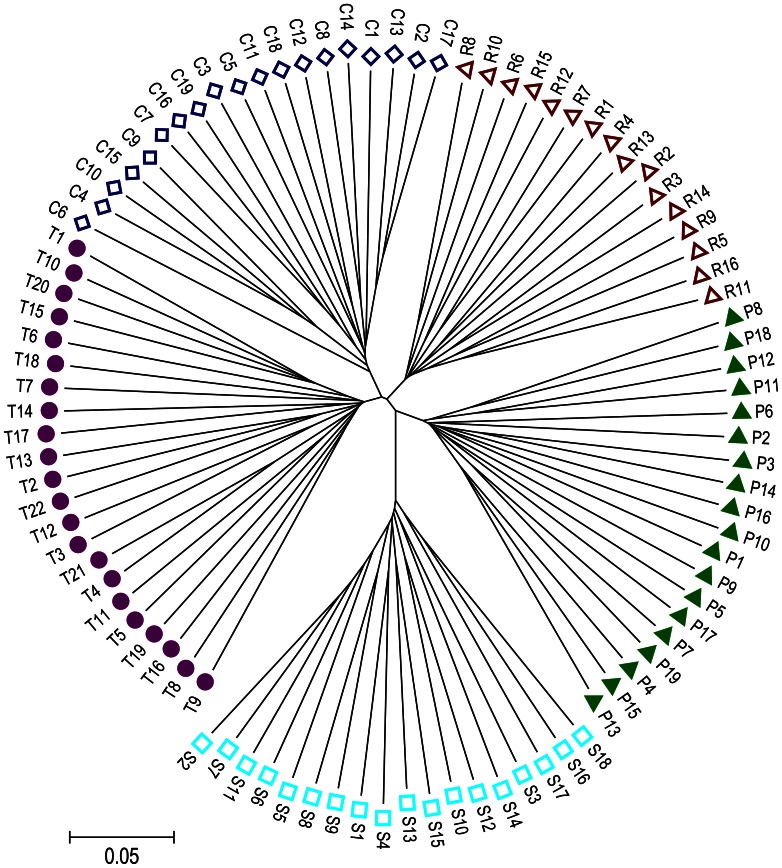
Neighbor-Joining tree relating the 94 individuals. The tree was constructed using allele sharing distances averaged over 46,850 SNPs. Different colors in labels represent the origin of breed individuals. S, R, C, P, and T represent Suffolk, Rambouillet, Columbia, Polypay, and Targhee sheep breeds, respectively. The meanings of S, R, C, P, and T are same in the following figures.

Figure 1 demonstrates a neighbor-joining (NJ) tree based on allele sharing distances (ASD) among 94 rams derived from Columbia, Polypay, Rambouillet, Suffolk, and Targhee breeds. The results clearly showed that there were no conflicts about the origin of individuals assigned to each breed. Also, the individuals from different sheep breeds were clearly clustered with closer genetic distances observed among Targhee, Columbia, Rambouillet, and Polypay breeds as compared to the Suffolk population.

### Accessing the Genetic Structure at the Population Level

As shown in Figure S3, the gene diversity, heterozygosity, and polymorphism information content (PIC) among five sheep populations were 0.3291–0.3576, 0.3496–0.3722 and 0.2619–0.2837 respectively. Polypay and Targhee populations had the highest gene diversity, heterozygosity, and PIC while the Suffolk population had the lowest values in these indexes. Classical F-statistics showed that most variation originated from individuals within a breed, while only 11% of the variation resulted from different breeds (Table S1). In particular, Targhee sheep had the highest within-breed variation.

**Figure 2:**
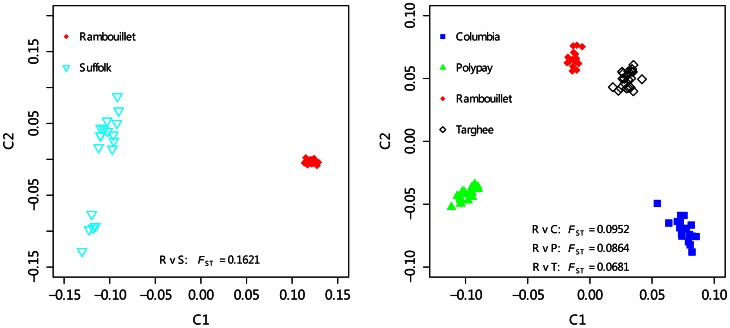
Multidimensional scaling plots for the genetic differentiations between Suffolk and Rambouillet (left) and among four Rambouillet-related breeds (right). *F*_ST_ represent the pair-wise *F*_ST_ between any two sheep breeds.

Furthermore, a multidimensional scaling plot clearly showed the genetic origin of breed between Suffolk and Rambouillet and among Rambouillet-related sheep breeds (Figure 2). Based on pair-wise estimates of *F*_ST,_ significant genetic differentiation appeared between Suffolk (meat breed) and Rambouillet (fine wool breed) (*F*_ST_ = 0.1621). In comparison, Rambouillet-related breeds were not significantly separated (*F*_ST_ = 0.0681–0.0952). In particular, Rambouillet and Targhee had the closest relationship (*F*_ST_ = 0.0681) (Figure 2).

### Population Differences in Minor Allele Frequencies

Based on minor allele frequency of SNPs, different levels of variation across breeds were observed. As shown in Figure S4, over 80% of the SNPs with one allele in all breeds had a MAF > 0.10. In all MAF ranges, the proportion of loci were significantly different among all sheep breeds (χ^2^ = 204.1084–1510.9140, *P* = 0.0000), indicating that each sheep breed had different numbers of SNPs in each MAF range.

### Characterization of DSRs between Suffolk and Rambouillet

**Figure 3:**
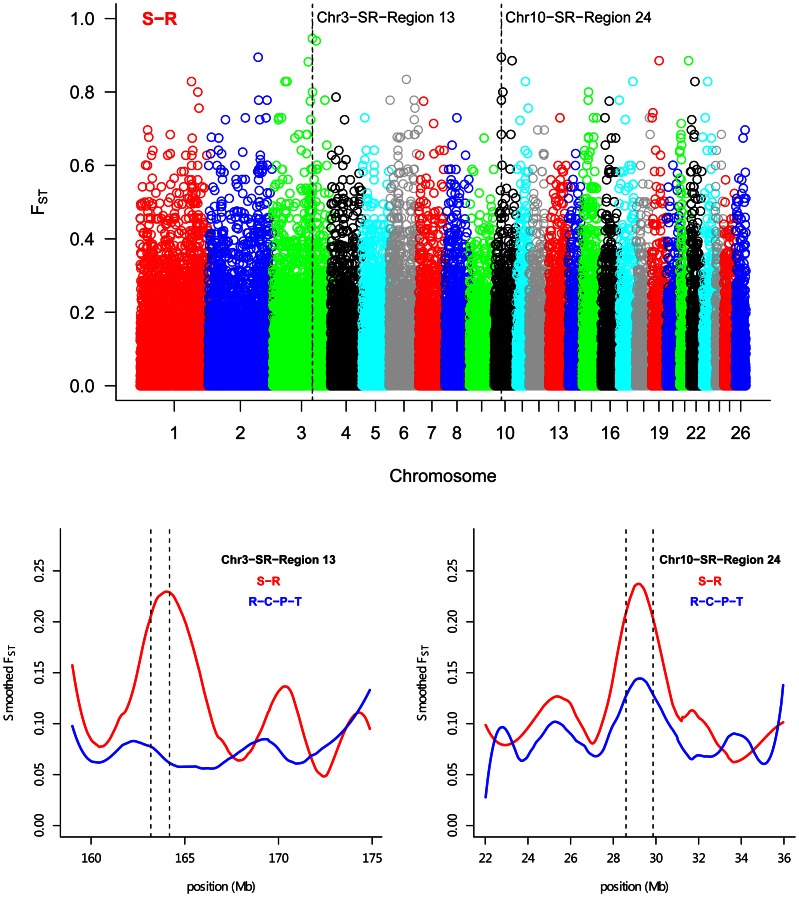
Genome-wide distribution of *F*_ST_ between Suffolk and Rambouillet. Based on OvineSNP50 BeadChip position, smoothed *F*_ST_ show that strong selection signals are observed in regions 13 and 24. S-R represents Suffolk-Rambouillet, while R-C-P-T means Rambouillet-Columbia-Polypay-Targhee.

Based on the SNP *F*_ST_ estimates, a total of 45 DSRs were identified in genomes between Suffolk and Rambouillet, which contained the top 0.1% of markers (48 SNPs in autosomal chromosomes and 6 SNPs in chromosome X; Table 1). Further examination of these DSRs identified 608 unique known genes, including 507 from autosomal DGRs and 101 from X chromosome DSRs (Table S2). The GO analysis revealed pathways enriched for a wide range of biological processes, such as regulation of organelle/cytoskeleton organization, translational elongation, protein catabolic processes, and cilium morphogenesis (Table S3). Among these 45 DSRs between Suffolk and Rambouillet, 13 also appeared as DSRs in cattle (Table S4). Among autosomal DSRs, the highest *F*_ST_ signal (OAR3_163921101, *F*_ST_ = 0.95, region 13) (Table 1 and Figure 3) was located at 153.26 Mb on ovine chromosome 3 (Ovine v3.1 genome), where glutamate receptor interacting protein 1 (GRIP1) resides. On the other hand, the DSR (named region 24, spans 28.58 to 29.84 Mb) with the highest number of top 0.1% SNPs was located on chromosome 10 (Table 1 and Figure 3). Furry homolog (Drosophila) (*FRY*), which included four of the top 0.1% SNPs of this region, is an evolutionarily conserved protein implicated in cell division and morphology [16]. Additionally, selection signals were detected for genes associated with economically important traits, i.e., *MITF* and *GHR*.

**Table 1:** Differential Genomic Regions between Suffolk and Rambouillet.

Region	Chr	OvineSNP50 BeadChipPosition (Mb)	Ovine version 3.1Position (Mb)	Peak SNP (*F*st)	Top0.1%	Top5%	Gene	Candidates
1	1	223,692,105.224,089,918	207,151,194. 207,545,071	OAR1_224053727 (0.83)	1	3	0	NA
2	1	250,742,595.252,518,595	232,582,008. 233,998,801	OAR1_251993749 (0.80)	1	7	3	NA
3	1	257,381,496.260,183,641	238,188,431. 240,583,116	OAR1_258333225 (0.76)	1	4	6	NA
4	2	202,599,712.203,260,925	191,179,157. 191,834,956	OAR2_202880426 (0.89)	1	5	3	NA
5	2	204,712,172.204,901,274	193,176,635. 193,374,050	OAR2_204724536 (0.78)	1	3	1	NA
6	2	238,542,709.240,388,073	225,842,690. 227,694,352	OAR2_23999476 7(0.78)	1	1	3	NA
7	3	26,522,262.27,337,380	24,478,117. 25,283,037	s53615 (0.73)	1	3	1	NA
8	3	36,636,298.39,008,465	34,218,962. 36,296,085	s07478 (0.73)	1	1	29	*PLB1*
9	3	51,184,039.53,141,887	48,017,454. 50,434,716	OAR3_51654982 (0.83)	1	1	10	*HSPA4*
10	3	59,599,168.61,257,663	56,387,458. 57,906,999	OAR3_59779674 (0.83)	1	2	24	NA
11	3	146,832,060.147,380,630	137,368,282. 137,726,913	OAR3_147229298 (0.88)	1	2	2	NA
12	3	154,771,125.155,555,840	144,946,795. 145,685,656	OAR3_154940822 (0.78)	1	4	3	NA
13	3	163,186,840.164,185,125	152,644,200. 153,519,437	OAR3_163921101 (0.95)	2	7	4	*GRIP1,HELB*
14	3	179,997,804.181,039,892	167,562,727. 168,548,617	OAR3_180711543 (0.94)	1	4	10	*ANKS1B*
15	3	216,437,540.217,530,305	201,198,665. 202,134,428	OAR3_217179573 (0.78)	1	3	11	*HEBP1*
16	4	22,765,057.24,779,248	21,632,745. 23,648,876	OAR4_23178649 (0.79)OAR4_23188917 (0.79)	2	3	3	NA
17	5	15,497,764.16,376,239	13,268,366. 14,154,057	s48780 (0.73)	1	4	31	*ARHGEF18*
18	6	7,001,684.8,507,164	5,094,411. 6,363,162	OAR6_7822475 (0.78)	1	2	6	NA
19	6	74,570,690.77,508,495	68,044,077. 71,068,886	s32922 (0.83)	1	0	18	NA
20	6	109,205,577.109,456,098	99,193,263. 99,428,191	OAR6_109386725 (0.78)	1	3	4	NA
21	6	111,022,637.112,892,917	100,900,651. 102,765,374	s61454 (0.76)	1	4	15	*PPP2R2C*
22	7	18,503,023.20,430,110	17,777,251. 19,581,841	OAR7_18600852 (0.78)	1	2	12	*THSD4*
23	8	47,992,613.49,442,593	44,527,752. 45,986,304	OAR8_48260342 (0.73)	1	2	0	NA
24	10	28,598,904.29,867,192	28,584,727. 29,842,383	OAR10_29223007 (0.89)OAR10_29341212 (0.89)	4	8	9	*FRY*
25	10	34,081,682.36,999,163	33,723,894. 36,238,012	DU468275_284 (0.80)	1	0	22	*SGCG*
26	10	80,478,253.82,187,881	73,523,887. 75,018,763	OAR10_80709863 (0.89)	1	1	7	NA
27	11	49,315,455.51,153,763	46,334,232. 48,164,176	s53904 (0.83)	1	5	32	NA
28	11	62,887,032.64,089,979	58,151,039. 59,339,383	OAR11_63882013 (0.76)	1	3	0	NA
29	15	28,713,885.31,235,158	27,396,963. 29,749,473	s10361 (0.80)	3	6	54	*TMPRSS4,* *PVRL1*
30	15	55,184,101.56,938,649	50,486,417. 51,939,057	s34973 (0.73)	1	2	18	NA
31	16	15,458,830.17,547,585	14,215,465. 15,879,198	OAR16_16902918 (0.73)	1	1	11	*RNF180*
32	16	32,916,237.34,666,518	30,290,785. 31,926,643	OAR16_34620156 (0.78)	1	4	12	*GHR*
33	17	172,595.2,807,057	47,789. 2,338,716	s47560 (0.78)	1	2	9	NA
34	17	64,389,126.64,669,860	58,960,116. 59,261,632	OAR17_64627979 (0.83)	1	2	0	NA
35	19	1,721,354.4,144,231	1,700,469. 3,900,485	OAR19_2610848 (0.73)	1	2	7	NA
36	19	5,578,148.5,861,149	5,354,385. 5,635,464	OAR19_5820545 (0.74)	1	2	0	NA
37	19	31,917,811.33,355,170	30,269,881. 31,674,797	OAR19_33278780 (0.89)	1	1	2	*MITF*
38	21	47,142,810.49,259,843	42,654,067. 44,580,177	OAR21_47788299 (0.89)	1	1	73	*OVOL1*
39	22	25,234,645.28,159,147	21,379,260. 23,858,085	OAR22_26729825 (0.83)	1	0	46	*CNNM2*
40	23	14,719,599.17,399,614	13,512,270.16,274,959	OAR23_15999547 (0.73)	1	1	3	NA
41	23	25,829,771.28,768,759	24,678,908. 27,575,686	OAR23_27272887 (0.83)	1	0	16	NA
42	X	51,771,664.53,703,822	51,193,144. 53,231,464	OARX_52914005_X (1.00)	2	2	35	*SHROOM4*
43	X	60,238,540. 64,779,887	56,827,620. 61,295,149	OARX_63571789 (1.00)	2	6	42	*MED12* *FAM155B*
44	X	97,956,675.99,324,958	77,903,961.79,702,349	S47111 (0.78)	1	2	18	NA
45	X	103,934,729.106,069,046	83,501,497.85,670,129	OARX_105162278 (0.80)	1	1	6	NA

Note: A total of 45 genomic regions contained the top 0.1% of SNPs ranked using *F*_ST_ (48 SNPs in autosomal and 6 SNPs in chromosome X). The candidate genes are given within the SNPs of top SNP (0.1%) for each region.

### Characterization of DSRs among Rambouillet-related Breeds

**Figure 4:**
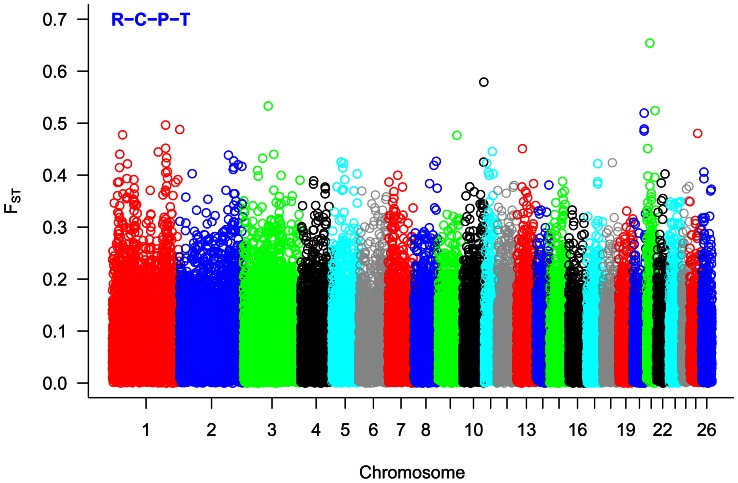
Genome-wide distribution of *F*_ST_ among Rambouillet, Columbia, Polypay and Targhee. R-C-P-T means Rambouillet-Columbia-Polypay-Targhee.

Genome-wide distribution of *F*_ST_ among Rambouillet, Columbia, Polypay, and Targhee are shown in Figure 4. Among these four Rambouillet-related breeds, 41 DSRs were identified with the top 0.1% of markers ranked by SNP *F*_ST_ (46 in autosomal and 1 in chromosome X, Table 2). These DSRs harbor a total of 526 unique genes, including 524 from autosomal DSRs and 2 from chromosome X DSRs (Table S5). Interestingly, GO analysis revealed that the enriched pathways were mainly related to cell adhesion processes (Table S6). Among these four sheep breeds, the OAR21_19719146 SNP (*F*_ST_ = 0.65, region 37) (Table 2) that belongs to potassium channel tetramerisation domain containing 14 (*KCTD14*) gene ranked highest, but unfortunately, little is known about this gene. Our data also show that both sheep and cattle may share eight DSRs identified among Rambouillet, Columbia, Polypay, and Targhee (Table S7).

**Table 2:** Differential Genomic Regions among Rambouillet, Columbia, Polypay and Targhee.

Region	Chr	OvineSNP50 BeadChip Position (Mb)	Ovine version 3.1 Position (Mb)	Peak SNP (*F*st)	Top 0.1%	Top 5%	Gene	Candidates
1	1	30,388,117.30,798,950	29,732,489. 30,143,388	OAR1_30552484 (0.41)	1	3	0	NA
2	1	31,470,501.32,656,978	30,814,041. 31,918,917	OAR1_31791056 (0.44)	1	2	6	*C10RF168*
3	1	43,609,775.44,153,575	42,128,684. 42,656,182	s54617 (0.48)	1	2	5	*IL23R*
4	1	65,172,267.67,951,620	61,589,657. 64,314,437	OAR1_66495276 (0.42)	1	1	24	*CLCA1*
5	1	202,259,180.203,821,806	187,627,690. 189,001,556	s48685 (0.44)	1	2	16	NA
6	1	234,341,600.234,884,834	217,261,740. 217,734,182	OAR1_234474464 (0.50)	3	4	3	*GOLIM4*
7	1	235,109,323.236,540,864	217,827,095. 219,332,381	OAR1_235893252 (0.42)	1	9	4	NA
8	1	238,519,182.238,893,868	221,199,906. 221,519,414	OAR1_238532462 (0.43)	1	2	0	NA
9	1	243,197,038.243,803,482	225,691,787. 226,307,550	OAR1_243366256 (0.41)	1	8	3	NA
10	2	52,036,310.54,944,048	48,501,546. 51,384,849	s67253 (0.40)	1	1	33	*TDRD7*
11	2	199,204,762.200,621,949	187,885,054. 189,186,475	OAR2_200574953 (0.44)	1	3	1	*CNTNAP5*
12	2	218,777,904.220,621,478	206,643,029. 208,373,192	OAR2_219736519 (0.41)	1	3	13	*ADAM23*
13	2	221,160,555.223,548,439	208,910,057. 211,274,257	s71750 (0.43)	2	6	16	NA
14	2	240,718,168.241,220,099	228,019,818. 228,451,875	OAR2_241048971 (0.42)	1	3	4	*COL4A4*
15	2	258,064,261.258,169,201	244,129,054. 244,237,213	s36316 (0.42)	1	2	0	NA
16	3	57,803,870.59,844,188	54,668,289. 56,584,259	OAR3_59312021 (0.41)	1	1	4	NA
17	3	79,812,234.81,533,150	75,538,807. 77,148,543	OAR3_79936157 (0.43)	1	2	17	NA
18	3	101,572,092.102,519,143	95,521,406. 96,371,620	s65088 (0.53)	1	2	32	*LOXL3*
19	3	124,569,037.125,411,739	116,863,473. 117,540,039	OAR3_125387325 (0.44)	1	3	3	NA
20	5	41,108,751.43,897,574	37,314,853. 40,020,855	OAR5_42607507 (0.43)	1	0	20	NA
21	5	50,423,296.51,450,144	46,337,373. 47,282,405	OAR5_50467325 (0.41)	1	2	18	NA
22	5	52,451,051.55,344,981	48,225,599. 51,058,240	OAR5_53870555 (0.42)	1	1	66	*PCDHB4*
23	5	110,241,515.110,742,508	101,265,722. 101,776,198	OAR5_110440932 (0.40)	1	2	0	NA
24	8	82,084,037.82,635,994	76,042,356. 76,469,230	s02095 (0.42)	1	2	4	NA
25	8	91,759,596.92,094,265	85,080,887. 85,410,438	s19702 (0.43)	1	3	1	NA
26	9	76,165,857.78,371,606	71,768,469. 73,808,972	OAR9_77597438 (0.48)	1	3	9	*RIMS2*
27	10	92,069,072.93,254,917	84,440,241. 85,596,458	DU434120_194 (0.58)	2	6	9	*ARHGEF7,* *TUBGCP3*
28	11	13,571,422.16,047,304	13,680,542. 15,833,930	OAR11_13845361 (0.42)	1	4	36	NA
29	11	21,539,612.22,907,346	20,832,059. 22,055,035	s50268 (0.41)	1	3	19	NA
30	11	42,268,122.42,641,540	39,778,473. 40,158,382	OAR11_42371614 (0.45)	1	3	16	NA
31	11	46,846,457.49,449,235	44,059,749. 46,470,862	s17065 (0.40)	1	4	33	NA
32	13	25,313,379.26,260,934	22,757,476. 23,728,650	OAR13_25941147 (0.45)	1	2	5	NA
33	17	53,094,617.53,266,879	48,796,359. 48,949,569	OAR17_53158609 (0.42)	1	3	1	NA
34	18	44,151,635.46,578,380	41,525,466. 43,758,521	OAR18_44175536 (0.42)	1	2	5	NA
35	20	45,499,537.49,145,527	41,875,844. 45,195,888	OAR20_47285319 (0.52)	3	0	22	NA
36	21	9,818,736.10,702,527	8,402,965. 9,308,676	s42617 (0.45)	1	3	8	*ME3*
37	21	18,653,273.20,194,612	16,451,437. 17,888,494	OAR21_19719146 (0.65)	1	5	16	*KCTD14*
38	21	46,857,187.48,167,168	42,551,316. 43,585,536	s67834 (0.52)	1	4	51	*BATF2*
39	25	31,163,834.32,984,881	29,829,111. 31,596,285	OAR25_32856981 (0.48)	1	2	12	NA
40	26	13,102,143.13,432,722	10,556,336. 10,883,077	OAR26_13165931 (0.41)	1	3	0	NA
41	X	112,364,319.112,895,373	107,382,577. 107,865,244	OARX_112730737 (0.44)	1	5	2	NA

Note: A total of 41 genomic regions contained the top 0.1% of SNPs ranked using *F*_ST_ (46 in autosomal and 1 in chromosome X). The candidate genes are given within the SNPs of top SNP (0.1%) for each region.

## Discussion

In the present study, we used the Illumina OvineSNP50 Genotyping BeadChip to analyze the genetic diversity and genome selection among Suffolk, Rambouillet, Columbia, Polypay, and Targhee sheep breeds from the USDA, ARS, U.S. Sheep Experiment Station. Our present study determined that close genetic relationships exist within Rambouillet-related breeds: Rambouillet, Columbia, Polypay, and Targhee, while Suffolk sheep are well separated from the Rambouillet-related breeds. The *F*_ST_ results showed significant genetic difference between Suffolk and Rambouillet (*F*_ST_ = 0.1621). Between these two distinct breeds, 45 DSRs and 608 candidate genes were identified using the Ovine Genome v3.1 Assembly as a reference. On the other hand, 41 DSRs and 526 genes were also determined among the four Rambouillet-related breeds.

Polypay (Finn × Targhee × Rambouillet × Dorset) [13], Targhee (Rambouillet × Lincoln × Corriedale), and Columbia (Lincoln × Rambouillet) are three breeds that were originally developed at the U.S. Sheep Experiment Station decades ago. Rambouillet and Suffolk were developed in France and England, respectively. In the present study, the highest gene diversity, heterozygosity and PIC were shown in Polypay and Targhee. This is not surprising because these two sheep breeds are the most recently developed breeds and we expect them to retain greater heterozygosity than the three other sheep breeds. Also, we found the *F*_ST_ averaged 0.1140 but Rambouillet-related breeds were not significantly separated, suggesting that the genetic differentiation is mainly between Suffolk and Rambouillet-related breeds. Not unexpectedly, cluster analysis also clearly showed that Suffolk is genetically distant from the other four sheep breeds (Figure 1). These results are rational because Columbia, Targhee, and Polypay are Rambouillet-related breeds. In particular, pair-wise *F*_ST_ estimation suggested the Targhee should be considered genetically most similar to Rambouillet (Figure 2). These results are reasonable because they are supported by our records and breed selection history of Columbia, Targhee, and Rambouillet. Recently, the same SNP chip was used to assign population of origin between wild sheep breeds, including bighorn and thinhorn sheep [9,] and to determine the historic selection of 74 sheep breeds [11]. And in cattle, the bovine SNP chip had also been used to reveal genetic history or population diversity [17]–[18]. Now, our results further confirm that the SNP chip is a powerful tool to discover the population genetic diversity in livestock and these data provided strong evidence of the genetic structure in these five sheep breeds.

Meat, wool, and dual-purpose breeds of sheep were developed because these are highly valued traits in sheep production. Based on genetic distance, our results indicated that the meat breed (Suffolk) is very distant from the fine wool breed (Rambouillet), which is very much in line with the functional purpose of the different breeds. For example, sheep selected for meat production generally have greater body weights. Mature weights of Suffolk rams, which have been historically selected for meat production, range from 113 to 159 kg and the fleece is considered a medium wool type with a staple length of 5 to 8.75 cm (http://u-s-s-a.org/). In comparison, mature Rambouillet rams, that have been bred to produce high quality wool, are smaller and weigh between 113 to 135 kg while the fleece staple length varies from 5 to10 cm and fiber diameter ranges from 18.5 to 24.5 microns (http://www.countrylovin.com/ARSBA/facts.htm).

In the present study, a genome-wide scan or differentiation analysis using *F*_ST_ revealed 45 chromosomal regions with evidence for selection. Interestingly, three regions, 19, 24, and 37, are almost identical to the regions identified in the 74 sheep breeds examined in [11], implying that important genomic selections might appear in these regions. Interestingly, region 24, which includes the *RXFP2* gene that is involved in horn morphology had a selection signal that was reconstituted only when comparing horned with polled populations [11], [19], was discovered in this study. Kijas et al (2012) [11] indicated this gene had the strongest selection signal due to the long-standing nature of selection. But in our study, only had two Rambouillet rams had horns., Therefore, the sample size was most likely too small to detect a difference in our study. Not unexpectedly, *GHR,* an important growth-related gene, was identified (region 32 on OAR 16). It is well-known that this gene affects body growth and decreases fatness [20], and its genetic variations are associated with growth traits in sheep or cattle [20]–[21]. Therefore, our study provides additional information for interpreting the difference in growth ability between Suffolk and Rambouillet. The highest ranked SNPs (*F*_ST_>0.90) were located in glutamate receptor interacting protein 1 (GRIP1) and ankyrin repeat and sterile alpha motif domain containing 1B (ANKS1B). Many studies have found GRIP1 plays an important role in receptor trafficking, synaptic organization, transmission in glutamatergic and GABAergic synapses and modulating autistic phenotype [22]–[24]. But unfortunately, little is known about the function of *GRIP1* in livestock. Here our results might provide a new clue for its role in sheep production. Recently, *ANKS1B* gene had been shown to be associated with body weight index and waist circumference in human GWAS studies [25], and Parker et al [26] indicated this gene may underlie the QTL associated with body weight in mice. Our studies also suggested *ANKS1B* gene might be a good growth trait candidate. However, additional studies are required to confirm this speculation. Interestingly, we discovered *MITF* gene in DSRs (region 37 on OAR 19). This gene accounts for pigmentation phenotypes in cattle [27]. However, *ASIP*, which controls a series of alleles of black and white coat color [28], was not included in our DSRs. In sheep, gene duplications might also cause black fleece [28]. In this study, the Suffolk has black head and legs, while the Rambouillet does have recessive black. It appears as though the key gene of pigmentation may provide evidence for selection between the two sheep breeds. In this study we also identified *FRY*, a gene involved in growing wing hairs [29] and bristles [30] in Drosophila. Mutations in *FRY* resulted in the formation of a strong multiple hair cell phenotypes that consisted of clusters of epidermal hairs and branched hairs [31]. But there is a little known about the role of *FRY* in livestock. In the present study, Rambouillet is often considered a fine wool breed while Suffolk has rather poor quality wool. Therefore, our results provide strong evidence for the role of *FRY* in sheep wool development. Additionally, 13 of the 45 DSRs identified in sheep represent those in cattle, suggesting that these genes are targets for selection across multiple species.

As described above, Columbia, Polypay, and Targhee are related to Rambouillet sheep. Among these four sheep breeds, a total of 41 DSRs were identified (Table 2). Interestingly, GO terms analyses of functionally known genes in these regions discovered pathways related to hemophilic/cell adhesion, translational elongation, germ cell development, sexual reproduction, and macromolecule biosynthetic processes. Some signature genes suggested strong selection given their roles, such as *CNTNAP5*, *ADAM23*, and *PCDHB4* in cell adhesion, *ME3, RIMS2,* and *TDRD7* in cellular respiration, cellular macromolecule/protein localization, and multicellular organism reproduction, respectively. These might result from long-term selection for improved reproduction and wool traits in these four sheep breeds [32]–[33].

In summary, we revealed the genetic diversity among Suffolk, Rambouillet, Columbia, Polypay, and Targhee sheep breeds of the United States using the Illumina OvineSNP50 BeadChip. Meanwhile, DSRs between Suffolk and Rambouillet and among Rambouillet-related sheep breeds were also identified with production of a list of candidate genes in these regions based on Ovine Genome v3.1 Assembly. Estimation of genome-wide diversity and identification of DSRs regions provide a powerful method to identify economically important trait-related genes that have been enriched during a long-term selection for different breeding objectives. Furthermore, our results also provide a foundation to further investigate sheep evolution and gene functions in the near future.

## Materials and Methods

### Ethics Statement

The U.S. Sheep Experiment Station Animal Institutional Care and Use Committee specifically approved this study (Protocol number: 11–01). All efforts were made to minimize any discomfort during blood collection.

### Sheep, DNA Preparation, and Genotyping on Illumina OvineSNP50 BeadChips

In the present study, blood samples were collected from 19 Columbia, 19 Polypay, 16 Rambouillet, 18 Suffolk, and 22 Targhee rams at the U.S. Sheep Experiment Station in Dubois, Idaho. Rams were produced from unique dams and 12, 12, 9, 10, and 17 unique sires of the Columbia, Polypay, Rambouillet, Suffolk, and Targhee breeds, respectively. The number of sheep per breed in this study is similar to the average number of sheep per breed Kijas and coworkers [11] used to quantify breed mixture and selection using the OvineSNP50 BeadChip. Blood was collected via jugular venipuncture into EDTA coated vacutainer tubes. Thereafter, DNA was extracted from 200 µL of whole blood with the GenElute Blood Genomic DNA extraction kit (Sigma, St. Louis, MO) according to the manufacturer’s instructions. All DNA samples were genotyped with standard procedures at GeneSeek (Lincoln, NE, US) on the OvineSNP50 genotyping BeadChip. Basic information on the 54,241 SNPs on the BeadChip, including SNP name, chromosome, and map location was provided by the service provider. The genotype quality control process was as previously described [34].

### Population Genetic Basics Analysis

Analysis of minor gene allele frequencies (MAF) was conducted with the chi-squared test using SAS Software for Windows v9.2 (SAS Institute Inc., Cary, NC). An exact test for Hardy-Weinberg Equilibrium (HWE) [35] of polymorphic SNPs was further carried out within each breed separately. We also computed the r^2^ measure between each marker pair within each breed separately using Haploview 4.1 [36]. Allele sharing distances of the neighbor-joining tree relating sheep individuals were computed by PowerMarker V3.25 software [37], and then the neighbor-joining tree was constructed by MEGA 5 [38].

Gene diversity, heterozygosity, polymorphism information content (PIC), and classical F-statistics [39] were calculated in the present study using PowerMarker V3.25. FSTAT 2.9.3.2 [40] was used to evaluate population relatedness using pair-wise estimates of *F*_ST_. An IBS matrix of distance (D) was constructed by Plink v1.07 [41], and then multidimensional scaling (MDS) analysis of 46,850 autosomal SNPs was determined using R 2.14.0 (www.r-project.org).

### Detection of Differentially Selected Regions (DSRs)

Fisher’s exact test was performed by R 2.14.0 to compare the allele frequencies between Suffolk and Rambouillet and among Rambouillet-related breed populations first. A SNP with a *P* value <0.05 was considered to be a statistically significant SNP after Bonferroni correction. Then, estimation of SNP and population-specific *F*_ST_ were based on the model proposed by Nicholson et al [42] and Flori et al [43]. The DSR algorithm was described previously [11], but with slight modifications: 1) raw values were ranked and used to identify regions; 2) the significant SNPs with 0.1% or 5% highest *F*_ST_ values were selected as the top significant SNPs; 3) centered on the top significant SNP (0.1%), neighboring markers were included until markers were encountered more than three consecutive SNPs ranking outside of the top significant 5%. We only considered the range between the upstream and downstream 1.5 Mb of the top SNP (0.1%) if the length of candidate regions were more than 3 Mb and combined any two regions as one region if they overlapped. SNP-specific *F*st values were smoothed over each chromosome with a local variable bandwidth kernel estimator [44].

Genes in these DSRs were examined for potential involvement in phenotypes using the Ovine Genome v3.1 Assembly (http://www.livestockgenomics.csiro.au/cgi-bin/gbrowse/oarv3.1/). The functional annotation of target genes for the gene ontology was performed using DAVID bioinformatics resources [45]. Allele frequency per breed for all DSR can be found at www.animalgenome.org/repository/pub/USDA2013.0411/.

## Supporting Information

Figure S1 **Distributions of SNPs on different chromosomes.**
(TIF)

Figure S2 **Decay of average pairwise r^2^ with inter-marker distance for the different sheep breeds.**
(TIF)

Figure S3 **Genetic diversity analysis in different sheep breeds.**
(TIF)

Figure S4 **Minor allele frequencies (MAF) with 46,850 SNPs for different sheep breeds.**
(TIF)

Table S1 Classical F-statistics in different sheep breeds.
(XLS)

Table S2 List of ovine positional candidate genes based on the predicted protein coding genes from DSRs between Suffolk and Rambouillet.
(XLS)

Table S3 Gene ontology analysis related to the ovine positional candidate genes from DSRs between Suffolk and Rambouillet.
(XLS)

Table S4 Selection signals identified in both sheep and cattle using the ovine positional candidate genes from DSRs between Suffolk and Rambouillet.
(XLS)

Table S5 List of ovine positional candidate genes based on the predicted protein coding genes from DSRs among Rambouillet, Columbia, Polypay, and Targee.
(XLS)

Table S6 Gene ontology analysis related to the ovine positional candidate genes from DSRs among Rambouillet, Columbia, Polypay, and Targhee.
(XLS)

Table S7 Selection signals identified in both sheep and cattle using the ovine positional candidate genes from DSRs among Rambouillet, Columbia, Polypay, and Targhee.
(XLS)
