# Supplementary figures and images for: Genome-Wide Genetic Diversity and Differentially Selected Regions among Suffolk, Rambouillet, Columbia, Polypay, and Targhee Sheep
Source: PLoS One. 2013 Jun 10;8(6):e65942. doi: 10.1371/journal.pone.0065942 (PMC3677876; doi:10.1371/journal.pone.0065942)

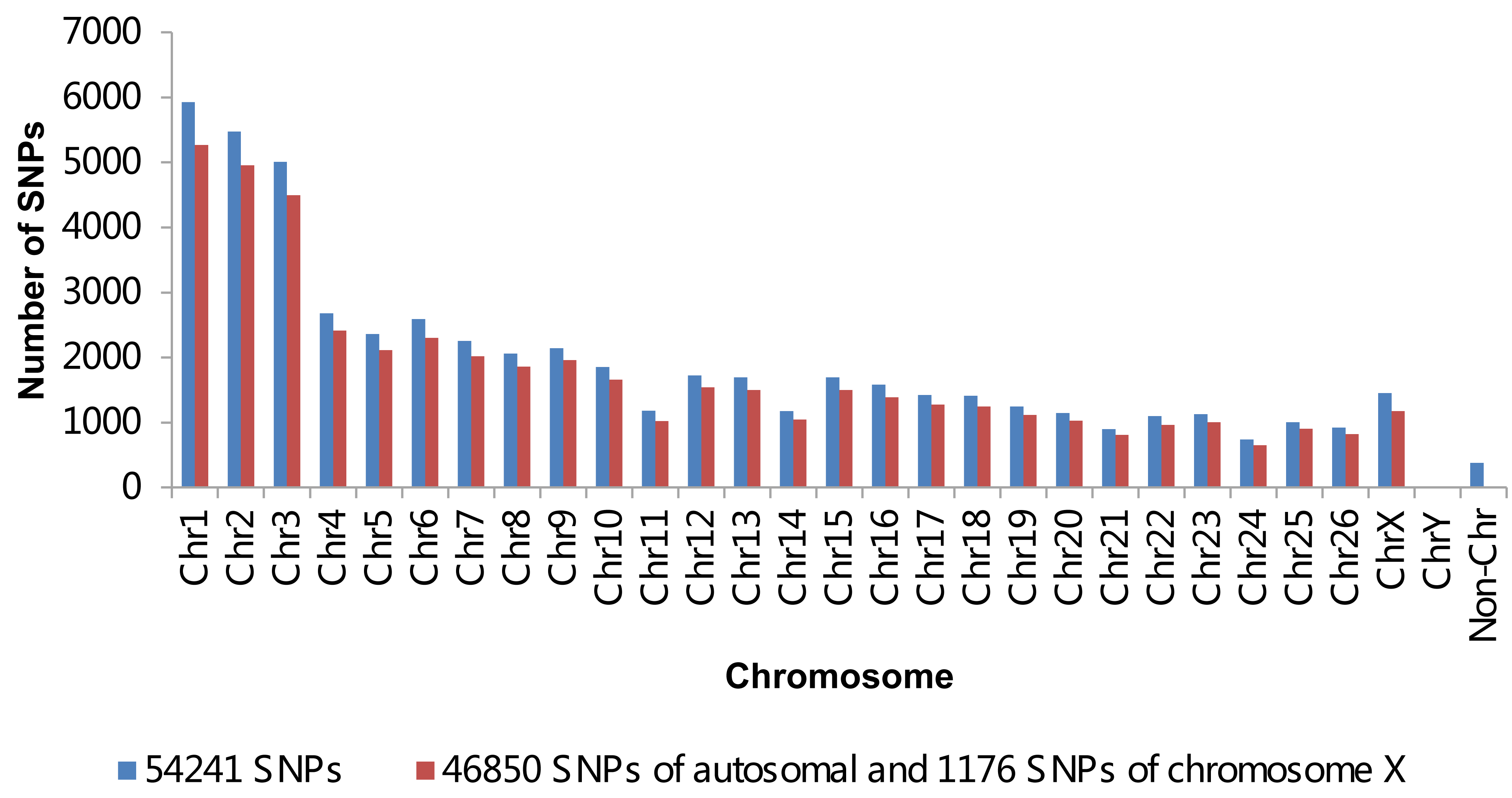

Supplement: Figure S1 — Distributions of SNPs on different chromosomes. (TIF) [file pone.0065942.s001.tif]

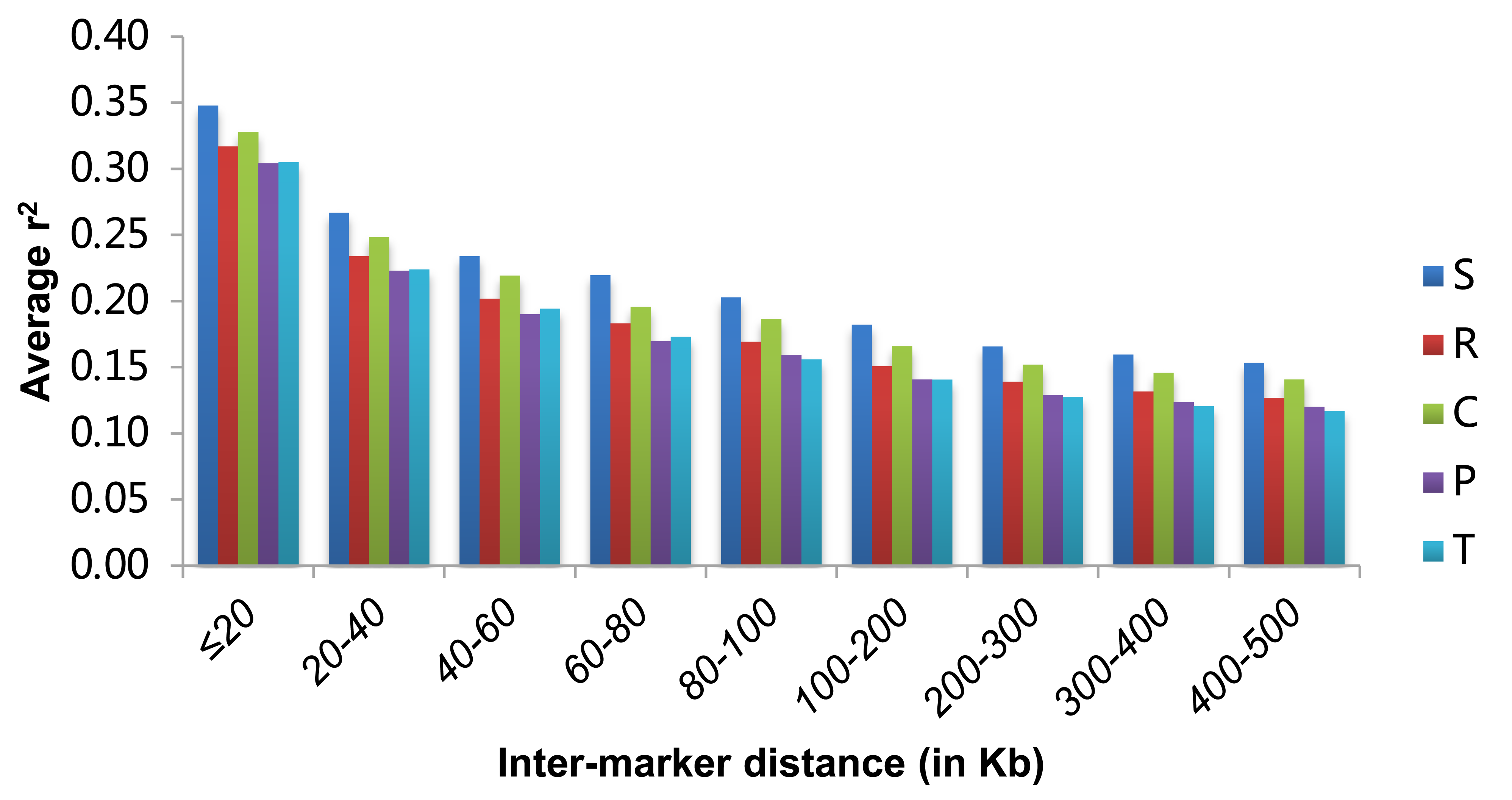

Supplement: Figure S2 — Decay of average pairwise r2 with inter-marker distance for the different sheep breeds. (TIF) [file pone.0065942.s002.tif]

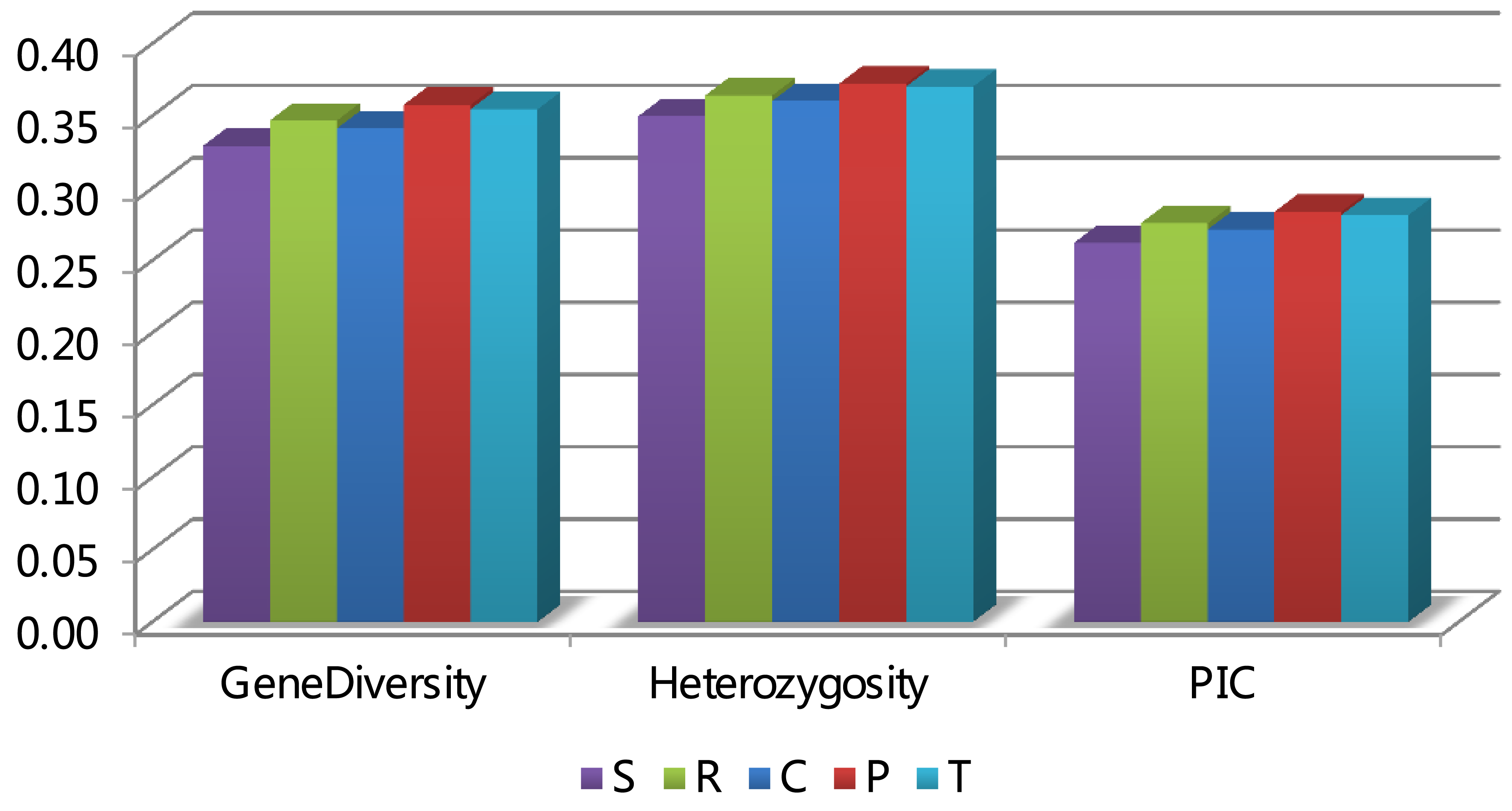

Supplement: Figure S3 — Genetic diversity analysis in different sheep breeds. (TIF) [file pone.0065942.s003.tif]

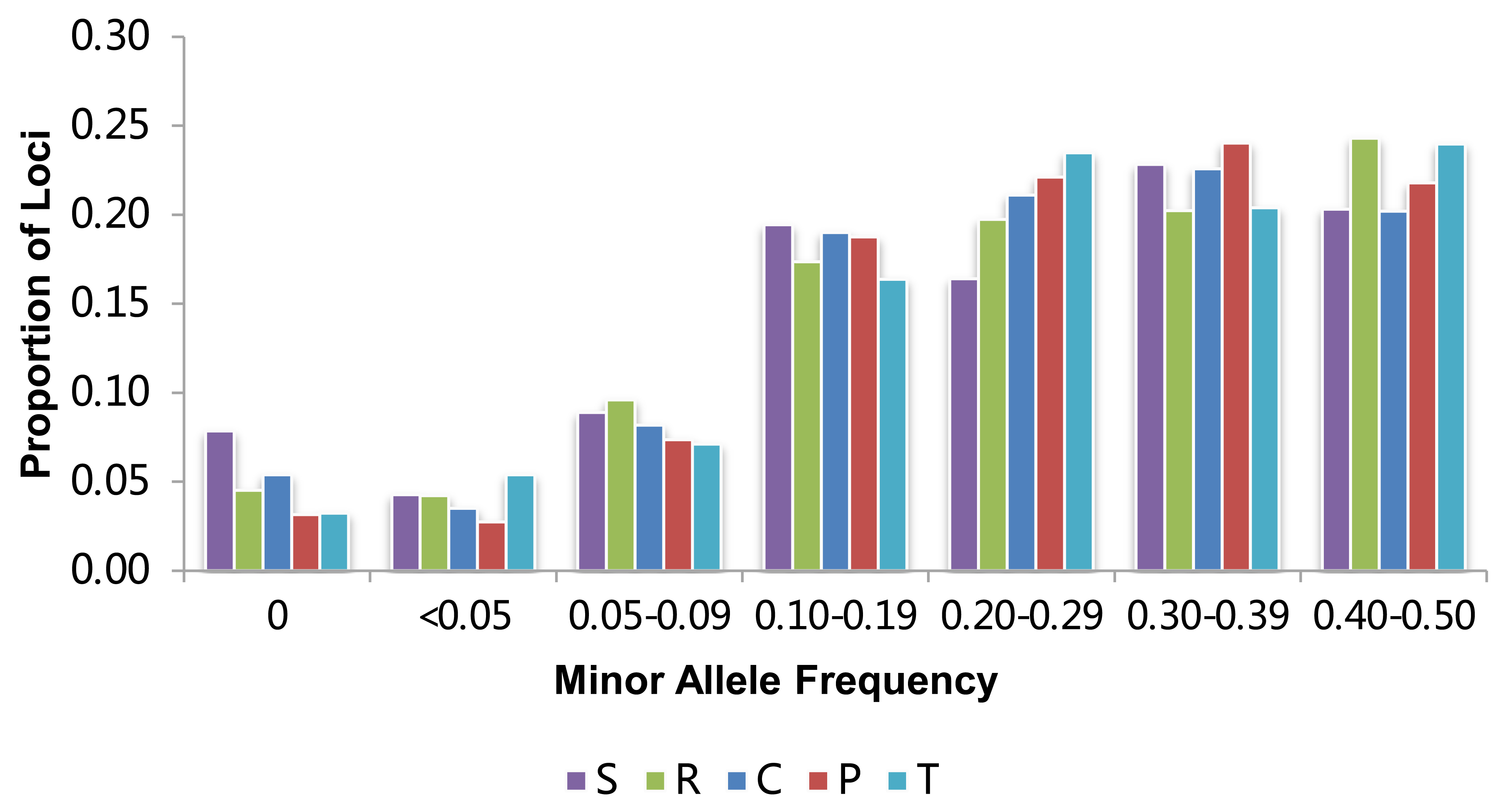

Supplement: Figure S4 — Minor allele frequencies (MAF) with 46,850 SNPs for different sheep breeds. (TIF) [file pone.0065942.s004.tif]
